# The genome sequence of bittersweet, *Solanum dulcamara* L. (Solanaceae)

**DOI:** 10.12688/wellcomeopenres.20004.1

**Published:** 2023-09-19

**Authors:** Maarten J. M. Christenhusz, Royal Botanic Gardens Kew Genome Acquisition Lab, Darwin Tree of Life Barcoding collective, Plant Genome Sizing collective, Wellcome Sanger Institute Tree of Life programme, Wellcome Sanger Institute Scientific Operations: DNA Pipelines collective, Tree of Life Core Informatics collective, Darwin Tree of Life Consortium

**Affiliations:** 1 Royal Botanic Gardens Kew, Richmond, England, UK

**Keywords:** Solanum dulcamara, bittersweet, genome sequence, chromosomal, Solanales

## Abstract

We present a genome assembly from an individual *Solanum dulcamara* (bittersweet; Eudicot; Magnoliopsida; Solanales; Solanaceae). The genome sequence is 946.3 megabases in span. Most of the assembly is scaffolded into 12 chromosomal pseudomolecules. The mitochondrial and plastid genomes have also been assembled, with lengths of 459.22 kilobases and 161.98 kilobases respectively.

## Species taxonomy

Eukaryota; Viridiplantae; Streptophyta; Streptophytina; Embryophyta; Tracheophyta; Euphyllophyta; Spermatophyta; Magnoliopsida; Mesangiospermae; eudicotyledons; Gunneridae; Pentapetalae; asterids; lamiids; Solanales; Solanaceae; Solanoideae; Solaneae; *Solanum,* *Solanum dulcamara* L. (NCBI:txid45834).

## Background

**Figure 1.**
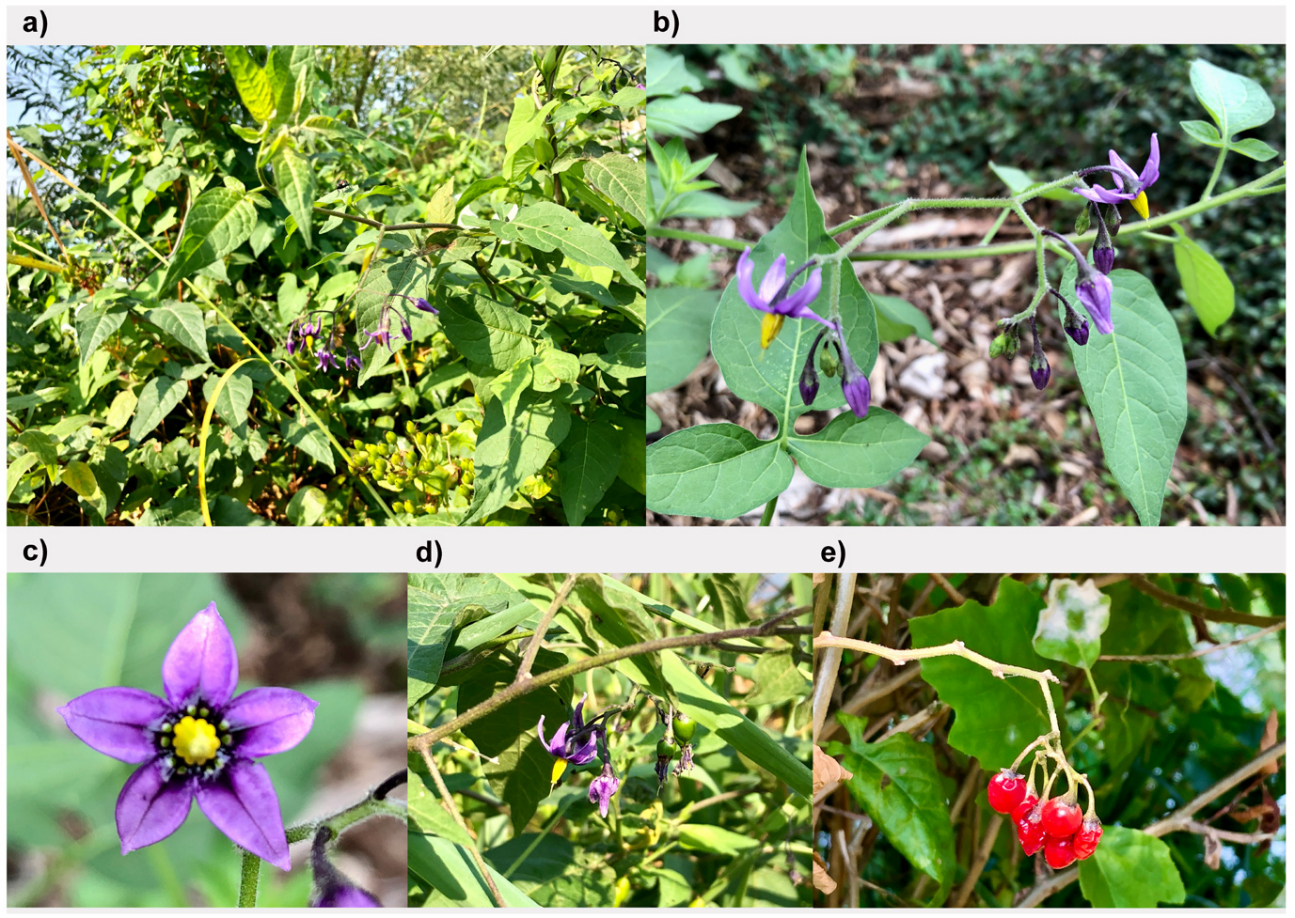
Photographs of the *Solanum dulcamara* (daSolDulc1) specimen used for genome sequencing. **a**. Habit. **b**, **d**. Inflorescence. **c**. Flower as seen from the front. **e**. Fruit.

Bittersweet, *Solanum dulcamara*, is a woody, perennial vine with foetid leaves. It has flowers with purple, reflexed petals and yellow stamens ( Figure 1). Pollinated by bees, it forms a bright red, ovoid berry that is dispersed by birds. It is found across Europe, north to Scandinavia and south to Greece and North Africa. It also grows throughout west and central Asia to Manchuria and has been introduced in North America. It occurs commonly across Britain and Ireland, but is rarer in the extreme north and west of the archipelago.

*Solanum dulcamara* is found in a variety of habitats, but it prefers moist places such as fens, marshes and lake and river shores, often periodically inundated. It can also be found in open woodland, hedgerows, and as a garden weed. The sample studied here originates from the shore of the River Thames in Kingston.

Bittersweet has been used by herbalists since ancient times in Europe to treat bruises and to fend off evil spirits ( Drage, 1665; Gerarde, 1597; Grieve, 1971). Its toxicity is relatively low, and poisoning is rare. The fruit is reported to be extremely bitter at first, followed by a sweet aftertaste, hence the name ( Mabey *et al.*, 1997).

It contains alkaloids that inhibit bacterial and tumorous growth ( Kumar *et al.*, 2009) and its antidermatophytic effect may be applied to treat ringworm and eczema ( Bakshi *et al.*, 2008; Fallahzadeh & Mohammadi, 2020).

The high-quality genome of *Solanum dulcamara* presented here complements the published chloroplast genome ( Amiryousefi *et al.*, 2018) and will be a useful resource for those studying Solanaceae and the distribution of alkaloids and medicinally useful compounds in this family.

## Genome sequence report

The genome was sequenced from a specimen of *Solanum dulcamara* ( Figure 1) collected from Kingston upon Thames, Surrey, UK (51.42, –0.31). Using flow cytometry, the genome size (1C-value) was estimated to be 1.24 pg, equivalent to 1,210 Mb. A total of 27-fold coverage in Pacific Biosciences single-molecule HiFi long reads and 66-fold coverage in 10X Genomics read clouds were generated. Primary assembly contigs were scaffolded with chromosome conformation Hi-C data. Manual assembly curation corrected 25 missing joins or misjoins and removed 15 haplotypic duplications, reducing the assembly length by 3.35% and the scaffold number by 26.71%, and increasing the scaffold N50 by 0.55%.

**Figure 2.**
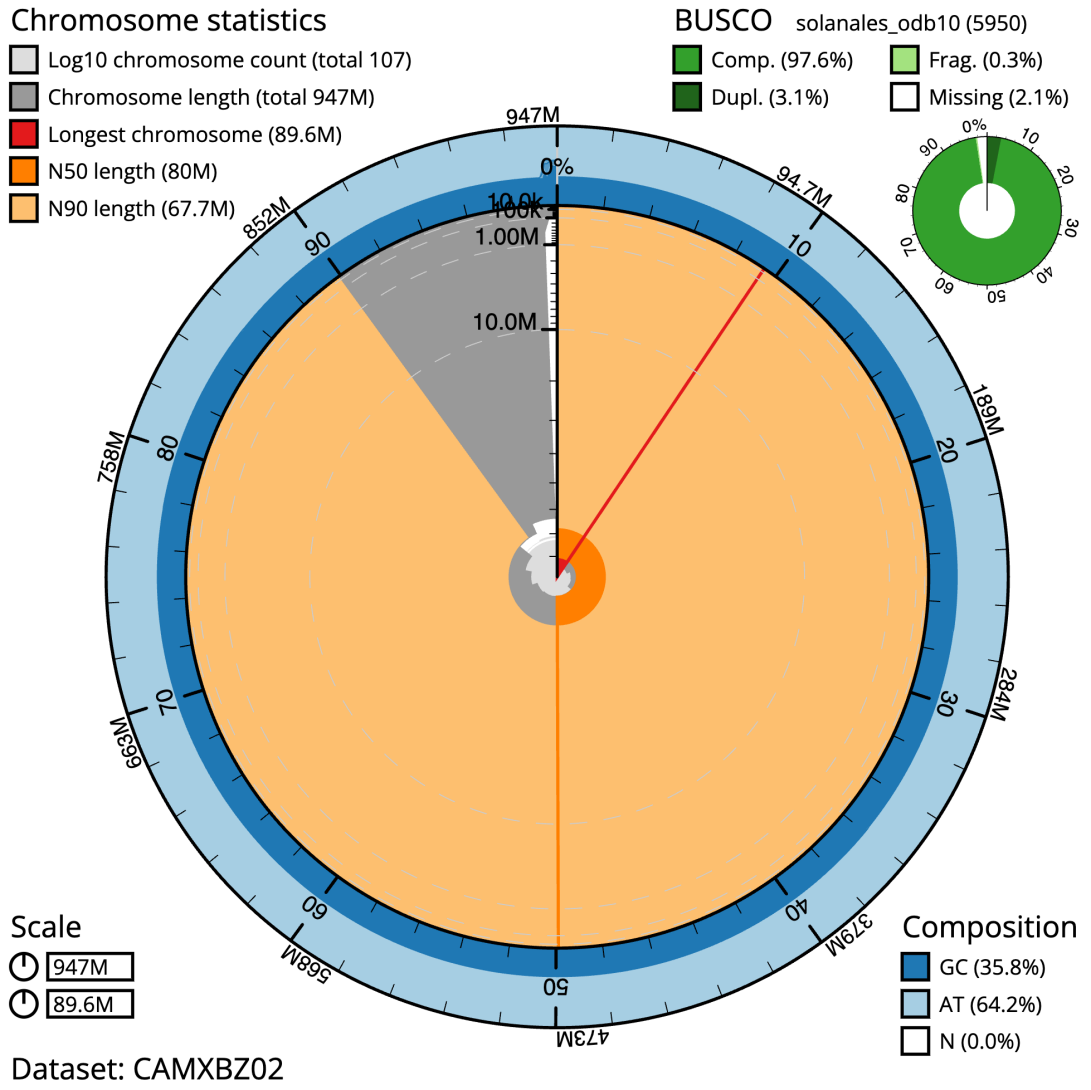
Genome assembly of *Solanum dulcamara*, daSolDulc1.2: metrics. The BlobToolKit Snailplot shows N50 metrics and BUSCO gene completeness. The main plot is divided into 1,000 size-ordered bins around the circumference with each bin representing 0.1% of the 946,926,110 bp assembly. The distribution of scaffold lengths is shown in dark grey with the plot radius scaled to the longest scaffold present in the assembly (89,582,784 bp, shown in red). Orange and pale-orange arcs show the N50 and N90 scaffold lengths (80,040,668 and 67,654,137 bp), respectively. The pale grey spiral shows the cumulative scaffold count on a log scale with white scale lines showing successive orders of magnitude. The blue and pale-blue area around the outside of the plot shows the distribution of GC, AT and N percentages in the same bins as the inner plot. A summary of complete, fragmented, duplicated and missing BUSCO genes in the solanales_odb10 set is shown in the top right. An interactive version of this figure is available at https://blobtoolkit.genomehubs.org/view/Solanum/dataset/CAMXBZ02/snail.

The final assembly has a total length of 946.3 Mb in 105 sequence scaffolds with a scaffold N50 of 80.0 Mb ( Table 1). Most (99.88%) of the assembly sequence was assigned to 12 chromosomal-level scaffolds. Chromosome-scale scaffolds confirmed by the Hi-C data are named in order of size ( Figure 2– Figure 5; Table 2). The order and orientation of scaffolds in repetitive regions on chromosome 6 (15.6 to 16.5 Mbp) are uncertain. Chromosome 10 shows an inversion between haplotypes (41 to 44 Mbp). While not fully phased, the assembly deposited is of one haplotype. Contigs corresponding to the second haplotype have also been deposited. The mitochondrial and plastid genomes were also assembled and can be found as a contig within the multifasta file of the genome submission.

The estimated Quality Value (QV) of the final assembly is 57.2 with *k*-mer completeness of 99.99%, and the assembly has a BUSCO v5.3.2 completeness of 97.6% (single = 94.5%, duplicated = 3.1%), using the solanales_odb10 reference set ( *n* = 5,950).

**Table 1.** Genome data for *Solanum dulcamara*, daSolDulc1.2.

Project accession data		
Assembly identifier	daSolDulc1.2	
Species	*Solanum dulcamara*	
Specimen	daSolDulc1	
NCBI taxonomy ID	45834	
BioProject	PRJEB47312	
BioSample ID	SAMEA7522044	
Isolate information	daSolDulc1, leaf (DNA sequencing, Hi-C scaffolding, RNA sequencing)	
Assembly metrics *		*Benchmark*
Consensus quality (QV)	57.2	*≥ 50*
*k*-mer completeness	99.99	*≥ 95%*
BUSCO **	C:97.6%[S:94.5%,D:3.1%], F:0.3%,M:2.1%,n:5,950	*C ≥ 95%*
Percentage of assembly mapped to chromosomes	99.88%	*≥ 95%*
Sex chromosomes	-	*localised homologous pairs*
Organelles	Mitochondrial and plastid genomes assembled	*complete single alleles*
Raw data accessions		
PacificBiosciences SEQUEL II	ERR6907982, ERR6907981	
10X Genomics Illumina	ERR6688683, ERR6688684, ERR6688681, ERR6688682	
Hi-C Illumina	ERR6688686	
PolyA RNA-Seq Illumina	ERR6688685	
Genome assembly		
Assembly accession	GCA_947179165.2	
*Accession of alternate haplotype*	GCA_947179135.2	
Span (Mb)	946.3	
Number of contigs	145	
Contig N50 length (Mb)	44.2	
Number of scaffolds	105	
Scaffold N50 length (Mb)	80.0	
Longest scaffold (Mb)	89.6	

* Assembly metric benchmarks are adapted from column VGP-2020 of “Table 1: Proposed standards and metrics for defining genome assembly quality” from ( Rhie *et al.*, 2021). ** BUSCO scores based on the solanales_odb10 BUSCO set using v5.3.2. C = complete [S = single copy, D = duplicated], F = fragmented, M = missing, n = number of orthologues in comparison. A full set of BUSCO scores is available at https://blobtoolkit.genomehubs.org/view/Solanum/dataset/CAMXBZ02/busco.

**Figure 3.**
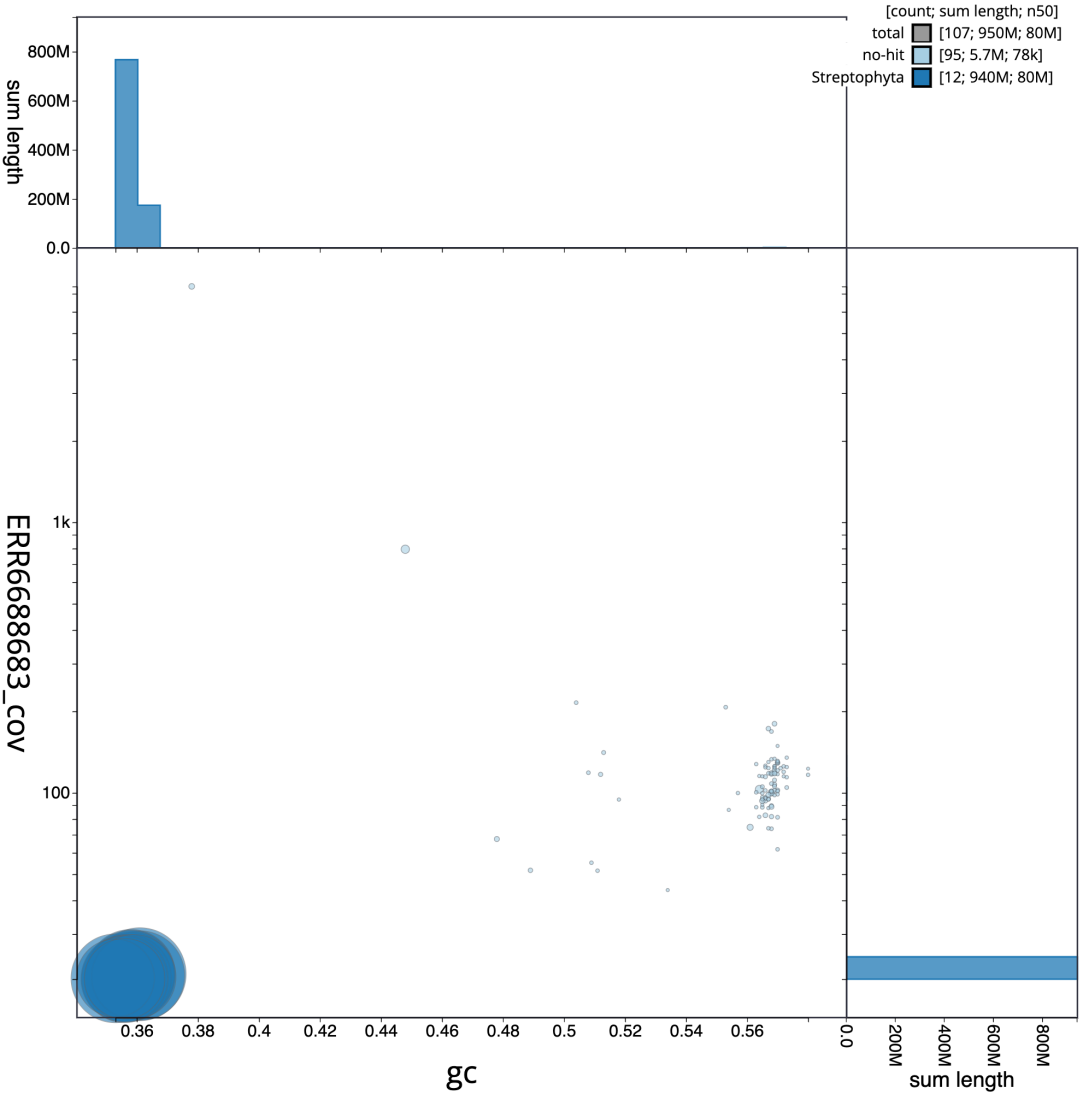
Genome assembly of *Solanum dulcamara*, daSolDulc1.2: BlobToolKit GC-coverage plot. Scaffolds are coloured by phylum. Circles are sized in proportion to scaffold length. Histograms show the distribution of scaffold length sum along each axis. An interactive version of this figure is available at https://blobtoolkit.genomehubs.org/view/Solanum/dataset/CAMXBZ02/blob.

**Figure 4.**
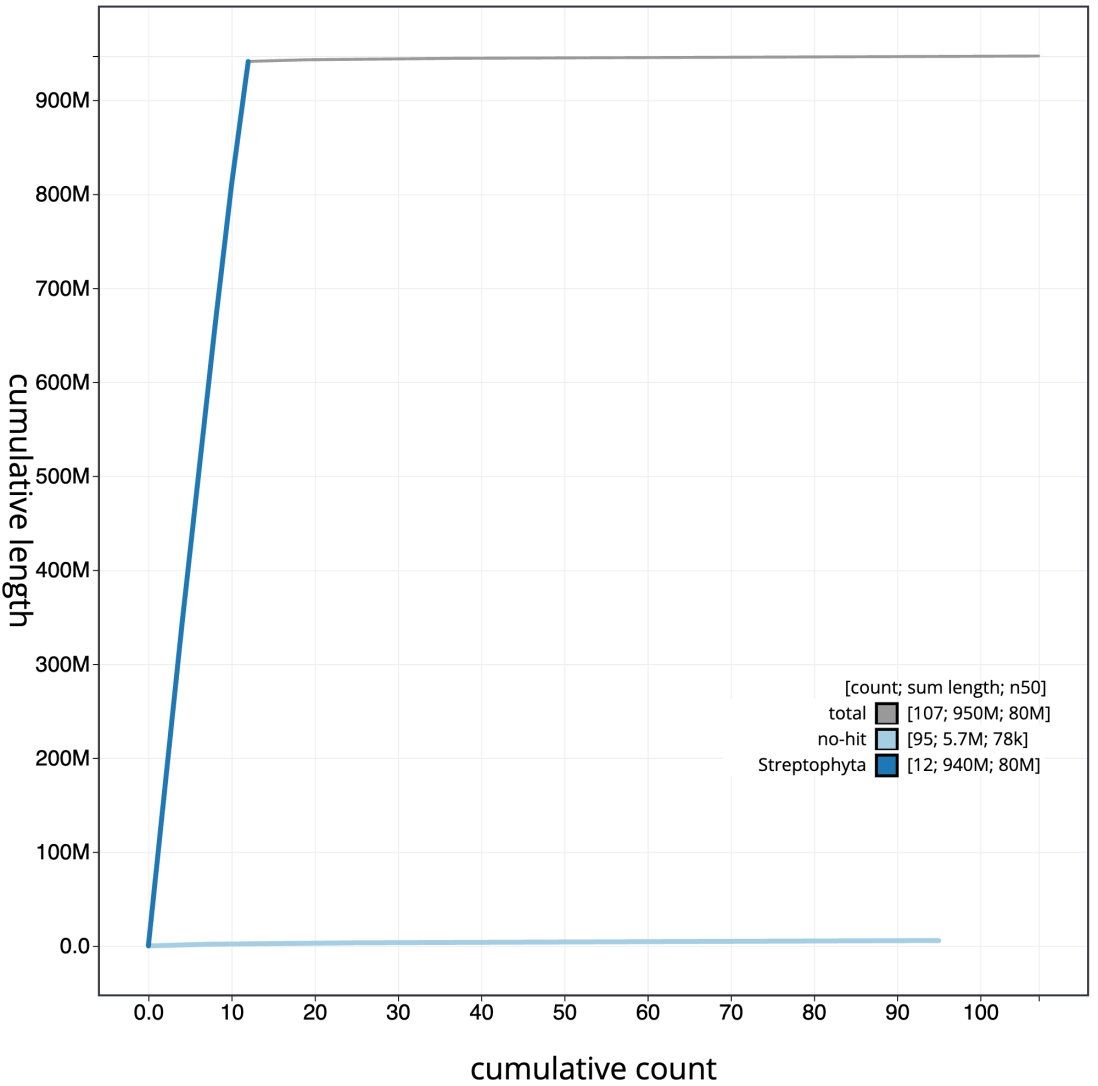
Genome assembly of *Solanum dulcamara*, daSolDulc1.2: BlobToolKit cumulative sequence plot. The grey line shows cumulative length for all scaffolds. Coloured lines show cumulative lengths of scaffolds assigned to each phylum using the buscogenes taxrule. An interactive version of this figure is available at https://blobtoolkit.genomehubs.org/view/Solanum/dataset/CAMXBZ02/cumulative.

**Figure 5.**
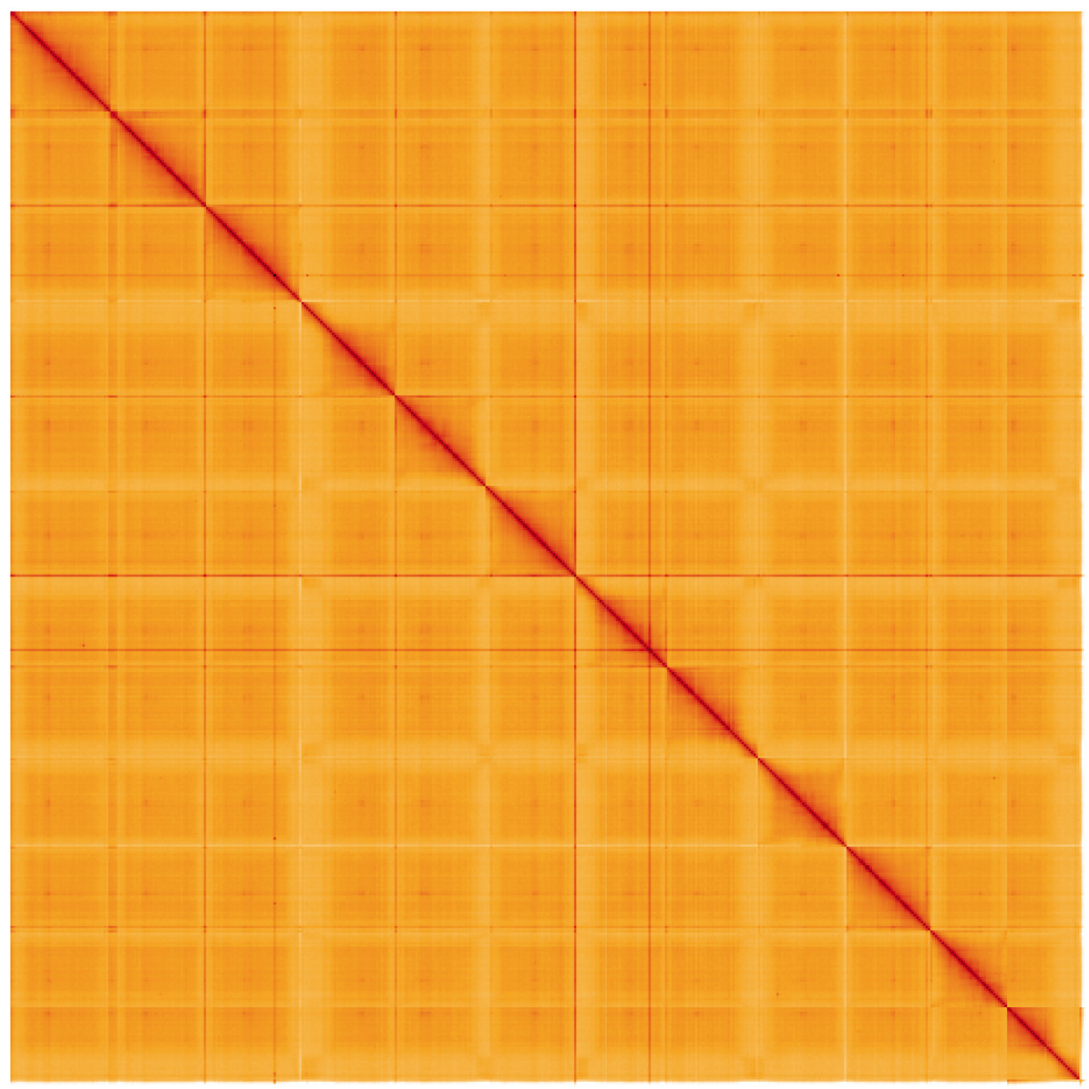
Genome assembly of *Solanum dulcamara*, daSolDulc1.2: Hi-C contact map of the daSolDulc1.2 assembly, visualised using HiGlass. Chromosomes are shown in order of size from left to right and top to bottom. An interactive version of this figure may be viewed at https://genome-note-higlass.tol.sanger.ac.uk/l/?d=aQnjuQF5TYGj41Ao0PQH1g.

**Table 2.** Chromosomal pseudomolecules in the genome assembly of *Solanum dulcamara*, daSolDulc1.

INSDC accession	Chromosome	Length (Mb)	GC%
OX359252.1	1	89.58	36.0
OX359253.1	2	84.29	36.0
OX359254.1	3	82.75	36.0
OX359255.1	4	82.47	35.5
OX359256.1	5	80.43	36.0
OX359257.1	6	80.04	36.0
OX359258.1	7	79.92	35.5
OX359259.1	8	79.48	35.5
OX359260.1	9	77.01	35.5
OX359261.1	10	74.23	36.0
OX359262.1	11	63.33	35.5
OX359263.1	12	67.65	35.5
OX381602.1	MT	0.46	45.0
OX381603.1	Pltd	0.16	38

Metadata for specimens, spectral estimates, sequencing runs, contaminants and pre-curation assembly statistics can be found at https://links.tol.sanger.ac.uk/species/45834.

## Methods

### Sample acquisition, genome size estimation and nucleic acid extraction

A *Solanum dulcamara* (specimen ID KDTOL10035, individual daSolDulc1) was picked by hand from Canbury Gardens, Kingston upon Thames, Surrey (latitude 51.42, longitude –0.31) on 2020-08-12. The specimen was collected and identified by Maarten J. M. Christenhusz (Royal Botanic Gardens, Kew) and was frozen at –80°C.

The genome size was estimated by flow cytometry using the fluorochrome propidium iodide and following the ‘one-step’ method as outlined in Pellicer *et al.* (2021). Specifically for this species, General Purpose Buffer (GPB) supplemented with 3% PVP and 0.08% (v/v) beta-mercaptoethanol was used for isolation of nuclei ( Loureiro *et al.*, 2007), and the internal calibration standard used was *Petroselinum crispum* ‘Champion Moss Curled’ with an assumed 1C-value of 2,200 Mb ( Obermayer *et al.*, 2002).

DNA was extracted at the Tree of Life laboratory, Wellcome Sanger Institute (WSI). The daSolDulc1 sample was weighed and dissected on dry ice with tissue set aside for Hi-C sequencing. The leaf sample was cryogenically disrupted to a fine powder using a Covaris cryoPREP Automated Dry Pulveriser, receiving multiple impacts. High molecular weight (HMW) DNA was extracted using the Qiagen Plant MagAttract DNA extraction kit. Low molecular weight DNA was removed from a 20 ng aliquot of extracted DNA using the 0.8X AMpure XP purification kit prior to 10X Chromium sequencing; a minimum of 50 ng DNA was submitted for 10X sequencing. HMW DNA was sheared into an average fragment size of 12–20 kb in a Megaruptor 3 system with speed setting 30. Sheared DNA was purified by solid-phase reversible immobilisation using AMPure PB beads with a 1.8X ratio of beads to sample to remove the shorter fragments and concentrate the DNA sample. The concentration of the sheared and purified DNA was assessed using a Nanodrop spectrophotometer and Qubit Fluorometer and Qubit dsDNA High Sensitivity Assay kit. Fragment size distribution was evaluated by running the sample on the FemtoPulse system.

RNA was extracted from leaf tissue of daSolDulc1 in the Tree of Life Laboratory at the WSI using TRIzol, according to the manufacturer’s instructions. RNA was then eluted in 50 μl RNAse-free water and its concentration assessed using a Nanodrop spectrophotometer and Qubit Fluorometer using the Qubit RNA Broad-Range (BR) Assay kit. Analysis of the integrity of the RNA was done using Agilent RNA 6000 Pico Kit and Eukaryotic Total RNA assay.

### Sequencing

Pacific Biosciences HiFi circular consensus and 10X Genomics read cloud DNA sequencing libraries were constructed according to the manufacturers’ instructions. Poly(A) RNA-Seq libraries were constructed using the NEB Ultra II RNA Library Prep kit. DNA and RNA sequencing was performed by the Scientific Operations core at the WSI on Pacific Biosciences SEQUEL II (HiFi), Illumina HiSeq 4000 (RNA-Seq) and NovaSeq 6000 (10X) instruments. Hi-C data were also generated from daSolDulc1 using the Arima2 kit and sequenced on the Illumina NovaSeq 6000 instrument.

### Genome assembly, curation and evaluation

Assembly was carried out with Hifiasm ( Cheng *et al.*, 2021) and haplotypic duplication was identified and removed with purge_dups ( Guan *et al.*, 2020). One round of polishing was performed by aligning 10X Genomics read data to the assembly with Long Ranger ALIGN, calling variants with FreeBayes ( Garrison & Marth, 2012). The assembly was then scaffolded with Hi-C data ( Rao *et al.*, 2014) using SALSA2 ( Ghurye *et al.*, 2019). The assembly was checked for contamination and corrected using the gEVAL system ( Chow *et al.*, 2016) as described previously ( Howe *et al.*, 2021). Manual curation was performed using gEVAL, HiGlass ( Kerpedjiev *et al.*, 2018) and Pretext ( Harry, 2022). The mitochondrial and chloroplast genomes were assembled using MBG from PacBio HiFi reads mapping to related genomes ( Rautiainen & Marschall, 2021). A representative circular sequence was selected for each from the graph based on read coverage.

A Hi-C map for the final assembly was produced using bwa-mem2 ( Vasimuddin *et al.*, 2019) in the Cooler file format ( Abdennur & Mirny, 2020). To assess the assembly metrics, the *k*-mer completeness and QV consensus quality values were calculated in Merqury ( Rhie *et al.*, 2020). This work was done using Nextflow ( Di Tommaso *et al.*, 2017) DSL2 pipelines “sanger-tol/readmapping” ( Surana *et al.*, 2023a) and “sanger-tol/genomenote” ( Surana *et al.*, 2023b). The genome was analysed within the BlobToolKit environment ( Challis *et al.*, 2020) and BUSCO scores ( Manni *et al.*, 2021; Simão *et al.*, 2015) were calculated.

Table 3 contains a list of relevant software tool versions and sources.

**Table 3.** Software tools: versions and sources.

Software tool	Version	Source
BlobToolKit	4.0.7	https://github.com/blobtoolkit/ blobtoolkit
BUSCO	5.3.2	https://gitlab.com/ezlab/busco
FreeBayes	1.3.1-17- gaa2ace8	https://github.com/freebayes/ freebayes
gEVAL	-	https://geval.org.uk/
Hifiasm	0.15.3-r339	https://github.com/chhylp123/ hifiasm
HiGlass	1.11.6	https://github.com/higlass/higlass
Long Ranger ALIGN	2.2.2	https://support.10xgenomics.com/ genome-exome/software/pipelines/ latest/advanced/other-pipelines
MBG	-	https://github.com/maickrau/MBG
Merqury	MerquryFK	https://github.com/thegenemyers/ MERQURY.FK
PretextView	0.2	https://github.com/wtsi-hpag/ PretextView
purge_dups	1.2.3	https://github.com/dfguan/purge_ dups
SALSA	2.2	https://github.com/salsa-rs/salsa
sanger-tol/ genomenote	v1.0	https://github.com/sanger-tol/ genomenote
sanger-tol/ readmapping	1.1.0	https://github.com/sanger-tol/ readmapping/tree/1.1.0

### Wellcome Sanger Institute – Legal and Governance

The materials that have contributed to this genome note have been supplied by a Darwin Tree of Life Partner. The submission of materials by a Darwin Tree of Life Partner is subject to the **‘Darwin Tree of Life Project Sampling Code of Practice’**, which can be found in full on the Darwin Tree of Life website here. By agreeing with and signing up to the Sampling Code of Practice, the Darwin Tree of Life Partner agrees they will meet the legal and ethical requirements and standards set out within this document in respect of all samples acquired for, and supplied to, the Darwin Tree of Life Project.

Further, the Wellcome Sanger Institute employs a process whereby due diligence is carried out proportionate to the nature of the materials themselves, and the circumstances under which they have been/are to be collected and provided for use. The purpose of this is to address and mitigate any potential legal and/or ethical implications of receipt and use of the materials as part of the research project, and to ensure that in doing so we align with best practice wherever possible. The overarching areas of consideration are:

•   Ethical review of provenance and sourcing of the material

•   Legality of collection, transfer and use (national and international) 

Each transfer of samples is further undertaken according to a Research Collaboration Agreement or Material Transfer Agreement entered into by the Darwin Tree of Life Partner, Genome Research Limited (operating as the Wellcome Sanger Institute), and in some circumstances other Darwin Tree of Life collaborators.

## Data Availability

European Nucleotide Archive: *Solanum dulcamara*. Accession number PRJEB47312; https://identifiers.org/ena.embl/PRJEB47312. ( Wellcome Sanger Institute, 2022) The genome sequence is released openly for reuse. The *Solanum dulcamara* genome sequencing initiative is part of the Darwin Tree of Life (DToL) project. All raw sequence data and the assembly have been deposited in INSDC databases. The genome will be annotated using available RNA-Seq data and presented through the Ensembl pipeline at the European Bioinformatics Institute. Raw data and assembly accession identifiers are reported in Table 1.

## Author information

Members of the Royal Botanic Gardens Kew Genome Acquisition Lab are listed here: https://doi.org/10.5281/zenodo.4786680.

Members of the Darwin Tree of Life Barcoding collective are listed here: https://doi.org/10.5281/zenodo.4893703.

Members of the Plant Genome Sizing collective are listed here: https://doi.org/10.5281/zenodo.7994306.

Members of the Wellcome Sanger Institute Tree of Life programme are listed here: https://doi.org/10.5281/zenodo.4783585.

Members of Wellcome Sanger Institute Scientific Operations: DNA Pipelines collective are listed here: https://doi.org/10.5281/zenodo.4790455.

Members of the Tree of Life Core Informatics collective are listed here: https://doi.org/10.5281/zenodo.5013541.

Members of the Darwin Tree of Life Consortium are listed here: https://doi.org/10.5281/zenodo.4783558.
