# Supplementary material for: The genome sequence of bittersweet, Solanum dulcamara L. (Solanaceae)
Source: Wellcome Open Res. 2023 Sep 19;8:409. [Version 1] doi: 10.12688/wellcomeopenres.20004.1 (PMC10585204; doi:10.12688/wellcomeopenres.20004.1)
Supplement: Peer review history

# Reviewer report: Reviewer response for version 1

**DOI:** 10.21956/wellcomeopenres.22150.r68509

**Published:** 2023-10-18

**Authors:** Xin Liu

**Affiliations:** 1 State Key Laboratory of Agricultural Genomics, BGI (Beijing Genomics Institute)-Shenzhen, Shenzhen, China

The data note by Christenhusz *et al.* described sequencing and genome assembly of bittersweet. The genome is relatively big, but the assembly is of high quality. The method is properly described and the genome assembly was properly assessed. The high quality genome dataset generated here can be valuable for future research.

Are sufficient details of methods and materials provided to allow replication by others?

Yes

Is the rationale for creating the dataset(s) clearly described?

Yes

Are the datasets clearly presented in a useable and accessible format?

Yes

Are the protocols appropriate and is the work technically sound?

Yes

Reviewer Expertise:

Genome assembly, plant genomics

I confirm that I have read this submission and believe that I have an appropriate level of expertise to confirm that it is of an acceptable scientific standard.

# Reviewer report: Reviewer response for version 1

**DOI:** 10.21956/wellcomeopenres.22150.r67452

**Published:** 2023-10-12

**Authors:** Jonathan Jones, Robert Heal

**Affiliations:** 1 University of East Anglia, Norwich, England, UK

This is a useful contribution to understanding the genomic diversity within the genus *Solanum *that carries ~ 1250 species including potato, tomato and aubergine.

 The authors should comment why there is such a large discrepancy between the 1C value for genome size of 1210Mb and the assembly size of 946Mb.

 The authors mention that they deposited sequence for one haplotype and that the two haplotypes differ by an inversion on chromosome 10. The authors should comment more on the extent of heterozygosity between the haplotypes, especially within all the genes and for all the BUSCO genes.

Are sufficient details of methods and materials provided to allow replication by others?

Yes

Is the rationale for creating the dataset(s) clearly described?

Yes

Are the datasets clearly presented in a useable and accessible format?

Yes

Are the protocols appropriate and is the work technically sound?

Yes

Reviewer Expertise:

Solanaceae as a source of novel disease resistance genes

We confirm that we have read this submission and believe that we have an appropriate level of expertise to confirm that it is of an acceptable scientific standard.
